# Contribution of Trimethylamine N-Oxide (TMAO) to Chronic Inflammatory and Degenerative Diseases

**DOI:** 10.3390/biomedicines11020431

**Published:** 2023-02-02

**Authors:** Luis A. Constantino-Jonapa, Yoshua Espinoza-Palacios, Alma R. Escalona-Montaño, Paulina Hernández-Ruiz, Luis M. Amezcua-Guerra, Amedeo Amedei, María M. Aguirre-García

**Affiliations:** 1 Unidad de Investigación UNAM-INC, División de Investigación, Facultad de Medicina, UNAM, Instituto Nacional de Cardiología Ignacio Chávez, Ciudad de México 14080, Mexico; 2 Departamento de Inmunología, Instituto Nacional de Cardiología Ignacio Chávez, Ciudad de México 14080, Mexico; 3 Department of Experimental and Clinical Medicine, University of Florence, 50134 Florence, Italy; 4 Interdisciplinary Internal Medicine Unit, Careggi University Hospital, 50134 Florence, Italy

**Keywords:** TMAO, microbiota, cardiovascular diseases, neurological diseases, metabolic diseases, COVID-19

## Abstract

Trimethylamine N-oxide (TMAO) is a metabolite produced by the gut microbiota and has been mainly associated with an increased incidence of cardiovascular diseases (CVDs) in humans. There are factors that affect one’s TMAO level, such as diet, drugs, age, and hormones, among others. Gut dysbiosis in the host has been studied recently as a new approach to understanding chronic inflammatory and degenerative diseases, including cardiovascular diseases, metabolic diseases, and Alzheimer’s disease. These disease types as well as COVID-19 are known to modulate host immunity. Diabetic and obese patients have been observed to have an increase in their level of TMAO, which has a direct correlation with CVDs. This metabolite is attributed to enhancing the inflammatory pathways through cholesterol and bile acid dysregulation, promoting foam cell formation. Additionally, TMAO activates the transcription factor NF-κB, which, in turn, triggers cytokine production. The result can be an exaggerated inflammatory response capable of inducing endoplasmic reticulum stress, which is responsible for various diseases. Due to the deleterious effects that this metabolite causes in its host, it is important to search for new therapeutic agents that allow a reduction in the TMAO levels of patients and that, thus, allow patients to be able to avoid a severe cardiovascular event. The present review discussed the synthesis of TMAO and its contribution to the pathogenesis of various inflammatory diseases.

## 1. Introduction

The last decade has seen a growing awareness of the importance of the host–microbiota relationship in human health. According to various studies, the microbiota plays a relevant role in several pathologies of growing concern to public health, including cardiovascular diseases, chronic kidney disease, and Alzheimer’s disease. Although it is not yet completely clear with which mechanisms the gut microbiota (GM) is implicated in the appearance and worsening of chronic inflammatory and degenerative diseases, among the possibilities under investigation is the role of some microbial metabolites. For example, trimethylamine N-oxide (TMAO) may markedly contribute to the exaggerated inflammatory response involved in the pathogenesis of the above-mentioned diseases. TMAO serves as an osmolyte and a metabolite in different microorganisms. It is a tertiary amine oxide resulting from the oxidation of the amino group of trimethylamines with a melting point of 95–99 °C, a solubility of 454 mg/mL in water and ethanol, and a molecular weight of 75.11 g/mol. Its molecular formula is C_3_H_9_NO [1].

The aim of the current review was to examine the evidence in the literature linking TMAO to chronic inflammatory and degenerative diseases.

## 2. TMAO Biosynthesis

**Figure 1:**
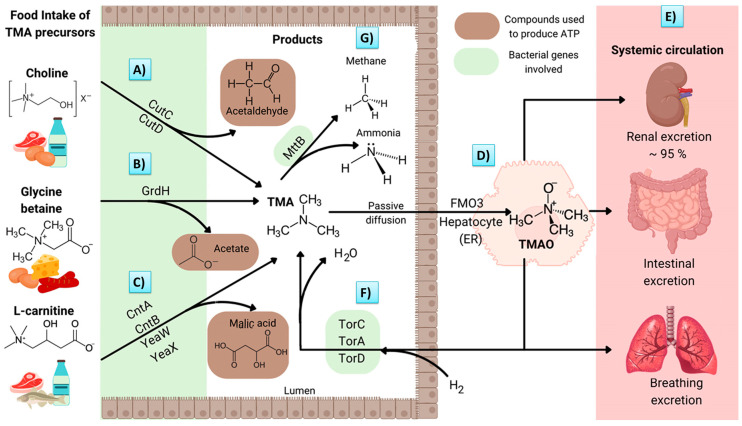
Illustration of the biosynthesis and metabolism of TMAO and the associated genes. Precursors of TMA, (**A**) choline, (**B**) glycine betaine, and (**C**) L-carnitine, are ingested in certain foods. They are then catabolized by the gut microbiota to obtain the end products (e.g., TMA) utilized in the formation of ATP. (**D**) Afterwards, 95% of TMA is diffused and oxidized by FMO3 in the liver; (**E**) subsequently, it is transported to other organs via systemic circulation. (**F**) TMAO can serve as a final electron acceptor in bacteria that colonize the gut, thus reducing TMAO to TMA and H_2_O. Alternatively, TMA is used by some methanobacteria to generate methane and ammonia. (**G**) Trimethylamine N-oxide is represented by TMAO, trimethylamine is represented by TMA, endoplasmic reticulum is represented by ER, and flavin-containing monooxygenase 3 is represented by FMO3. (Created with canva.com and BioRender.com).

One of several enzymes capable of promoting TMAO production in the intestinal tract is choline lyase, which is classified as an anaerobic glycyl radical enzyme. It is encoded by the *cutC* gene, which is activated by *cutD* [2]. The main function of this cluster is the production of acetyl-CoA and ATP (Figure 1A). 

Trimethylamine (TMA), a TMAO precursor, is generated by the bacterial genes *cntA* and *cntB*. The latter codes for an NAD (P)-dependent reductase, while *cntA* codes for a choline monooxygenase. Both enzymes use carnitine as a substrate for the degradation and synthesis of TMA and malic acid (Figure 1B) [3]. In addition, closely related to cntA and cntB, there are other similar genes, such as *yeaW* and *yeaX*, which code for a monooxygenase and a reductase, respectively. The functional domain of the *YeaW* monooxygenase is almost identical to that of the *cntA* monooxygenase, and, in collaboration with the *yeaX* reductase, it synthetizes TMA using γ–butyrobetaine as an intermediate [4].

In a third pathway for TMA synthesis, glycine betaine serves as the substrate. The *grdH* gene codes for a glycine betaine reductase, which is responsible for the reduction of glycine betaine to acetate and TMA. Subsequently, the acetate molecule is oxidized to provide electrons for betaine reduction (Figure 1C) [5]. 

A significant amount of TMA is synthetized by the gut microbiota and enters portal circulation through passive diffusion through the enterocytes. Upon reaching the liver via flavin-containing monooxygenase 3 (FMO3), approximately 95% of TMA is converted into TMAO (Figure 1D) [6].

There is still another pathway involving bacteria and TMAO. Under anaerobic conditions, TMAO is an electron acceptor for some bacteria species (e.g., *Escherichia coli*) that colonize the human intestine [7]. TMAO respiration proteins are encoded by the *torCAD* operon, which is composed of *torC* (the c-type cytochrome), *torA* (reductase), and *torD* (*torA*-chaperone) [8], which, in conjunction, reduce TMAO to TMA and H_2_O (Figure 1F). Apart from *E. coli*, this respiration system (mainly existing in marine species) is found in the *Klebsiella* and *Actinobacteria* species, which are sometimes present in the human gut microbiota [9], according to a metagenomic study.

A quick search of the UniProt database (www.uniprot.org) was carried out with the *torC* sequence from the K12 strain of *E. coli* (UniProt ID: P33226) as the input. The TMAO anaerobic respiration system is apparently widely distributed in the species of *Firmicutes, Actinobacteria*, *and Proteobacteria*, the main phyla associated with TMA production in the gut during the development of various diseases. Such species include *Vibrio, Salmonella, Shigella, Citrobacter, Hafnia, Serratia,* and *Haemophilus*. 

Although a relationship between the *torCAD* operon and human health has not been established, this respiration system could possibly be relevant in generating dysbiosis, a condition found in some pathologies (e.g., cardiovascular diseases), where TMAO plays a role. According to one study, TMAO respiration is involved in triggering the synthesis of the cholera toxin [10], suggesting that the expression of other proteins or bacterial toxins might be linked to the TMAO respiration system.

A new order of methanobacteria capable of colonizing the human gut was proposed in 2018 when *Methanomassiliicoccus luminyensis (M. luminyensis*) was isolated [11]. *M. luminyensis* may be able to deplete TMA through H_2_-reduction, which is undergone for methane production, and the catalyst of the reaction is predicted to be a methyltransferase encoded by *mttB* (Figure 1G) [12]. Hence, *M. luminyensis* could potentially be used as a probiotic to treat several diseases, leading to a decline in the serum TMAO concentration and a decrease in the associated risks. This bacterial species reportedly has certain characteristics of commensal gut microbes, including susceptibility to human-derived antimicrobial peptides and a low immunogenicity against human immune cells [13]. All the bacterial genes related to the metabolism of TMAO are shown in Table 1.

## 3. Factors Affecting the TMAO Level

### 3.1. Diet

Multiple factors have been found to influence one’s level of TMAO, including the type of intestinal microbiota and the diet of the individual. The consumption of eggs, beef, and/or fish increases one’s TMAO level due to the presence of the TMA precursors, choline, carnitine, and betaine. Additionally, a TMA precursor-rich diet is associated with an increase in the *Firmicutes–Bacteroidetes* ratio (the main *genera* associated with TMAO synthesis) and a lower gut microbiota diversity [14]. One study detected as much as a 20-fold greater TMAO production in omnivores versus vegans/vegetarians following L-carnitine consumption [15]. 

On the other hand, people whose diets are plant-based (vegetarian, vegan, or Mediterranean diets) have a significantly lower concentration of TMAO [16]. Thus, one’s diet is an important factor which determines his/her TMAO level and can be used as an intervention to decrease the TMAO level in patients with diseases such as cardiovascular diseases, type 2 diabetes, hypertension, Alzheimer’s disease, and others. For example, in murine models, dietary fiber was reported to decrease the TMAO level by 62.6%. It was proposed that fiber could activate the AMPK pathways, leading to a decline in the generation of TMA-lyase and other ATP-consuming processes [17].

### 3.2. Drugs

The level of TMAO can also be affected by some drugs. Statins, one of the most widely used groups of drugs for treating dyslipidemia, have been shown to reduce the serum TMAO concentration. The mechanism is hypothesized to be based on gut microbiota alterations [18,19]. 

Metformin, the main drug administered for the treatment of type 2 diabetes (T2D), might reduce the level of TMAO. Although one study found no changes in the TMAO level after 3 months of metformin consumption in humans [20], another detected a decrease in this metabolite in metformin-treated versus untreated patients [21]. Interestingly, a lower level of TMAO was observed in metformin-treated versus control db/db mice. According to the authors, metformin may inhibit TMA generation performed by the gut microbiota. Along the same line, *Klebsiella pneumonia* and *Proteus mirabilis* exhibited a lower in vitro production of TMA in the presence of metformin; currently, it is not yet fully understood since metformin does not alter the expression of TMA-producing enzymes or the uptake of choline. The activity of metformin appears to be more complex, probably involving another component of the *Cut* gene cluster [22].

Acetylsalicylic acid (aspirin) combined with choline supplementation has been reported to diminish the level of TMAO in prothrombotic patients [23]. As expected, the latter supplementation alone induces an increase in the TMAO level and, in addition, boosts platelet hyperreactivity, which is limited by the aspirin treatment. According to previous studies, aspirin alters the composition of the intestinal microbiota, where the abundance of *Prevotella* spp., *Bacteroides* spp., the *Ruminococcaceae* family, and *Barnesiella* spp. distinguishes patients who are aspirin consumers from those who are nonconsumers [24]. The fact that the *Prevotella, Bacteroides,* and *Barnesiella* species contain the *CutC* gene (known to encode an enzyme responsible for intestinal TMA synthesis) may explain the reason for this decrease in aspirin-treated patients. The mechanism of nonsteroidal anti-inflammatory drugs (NSAIDs) such as aspirin has been studied in animal models. The administration of indomethacin in mice caused intestinal inflammation, activating TLR2 and TLR4 and increasing the abundance of *Bacteroides, Enterobacteriaceae,* and *Clostridium* [25]. The intragastrical administration of aspirin increased oral microbial diversity in rats, increasing *Lactobacillaceae* in particular. In addition, aspirin reduced the IgG and IgA content in the saliva, contributing to an increase in microbial diversity [26]. Additionally, aspirin has been reported to be an inhibitor of *cutC* TMA-lyase, one of the enzymes responsible for TMA production and, thus, TMAO production [27]. As was mentioned in a previous section, bacteria that contain TMA-lyase can obtain energy from the cleavage of choline to TMA, which favors their growth. Inhibiting this enzyme can help to reduce the growth of the *cutC* gene-containing bacteria that are responsible for TMAO production.

Other drugs have been used to decrease the TMAO level by targeting TMA-lyase enzymes. Berberine, a drug from the plant *Coptidis Rhizoma*, has been used to treat patients with atherosclerosis, type 2 diabetes, and/or hyperlipidemia [28]. In rat models, it has been demonstrated that berberine reduces TMAO levels, directly inhibiting *cutC* choline-TMA-lyase [28]. Berberine is already being used in clinical studies in patients with atherosclerosis [28].

Another compound that inhibits *cutC* lyase is 3,3-Dimethyl-1-butanol (DMB), an analogue of choline that can be found in balsamic vinegar, red wine, olive oil, and grape seed oil [29]. This compound can reduce the TMAO levels in ApoE −/− mice with a choline-rich diet, decreasing foam cell formation and atherosclerotic lesions [29]. DMB also reduced the TMAO level in animal models of obesity, kidney disease, and aged individuals [30,31,32].

Meldonium is an aza-analogue of the carnitine bioprecursor γ-butyrobetaine, and it can reduce the TMAO level and is used as an antiatherosclerotic compound. One of the mechanisms proposed for the reduction of TMAO is *cntA* monooxygenase inhibition, the enzyme responsible for the cleavage of L-carnitine to TMA [33].

In recent years, new drugs have been developed for reducing TMAO production by targeting TMA-producing enzymes. Iodomethylcholine (IMC) and fluoromethylcholine (FMC) are inhibitors of *cutC* TMA-lyase, and they have a limited systemic exposure and a low toxicity, with a higher efficiency than DMB [34]. Both drugs reduced thrombosis and platelet responsiveness in mice and, therefore, can be used to treat patients with diseases such as cardiovascular diseases [34].

Table 2 shows the drugs/compounds that affect the level of TMAO.

### 3.3. Age

TMAO levels are higher in elderly individuals than in younger individuals [31]. The main factor that influences this increase in the TMAO levels of the elderly is the dysbiosis of the gut microbiota caused by aging. Aging is considered to be the progressive loss of homeostasis and metabolic functions. There are hallmarks of this process, but there are also changes in the gut microbiota composition [38]. These changes in the microbiota are influenced by the physiology of the gastrointestinal tract, such as inflammation, which leads to the onset of several diseases [39]. One of the factors that causes the changes in the gut microbiota is the decline of the immune system, which affects the relationship between the host and the microbiota, leading to dysbiosis [40] and increasing the *Firmicutes–Bacteroidetes* ratio and, thus, TMAO production, which is associated with an increase in this ratio [41]. In addition to host factors, the microbiota composition can be influenced by external factors, such as diet, medication, physical activity, etc. [42]. 

### 3.4. Hormones

Some hormones have a regulatory effect on TMAO production. In a mouse model, gonadectomized animals showed an enhanced expression of FMO3 and an elevated TMAO level, while dihydrotestosterone administration caused a decrease in the latter metabolite. An oophorectomy, on the other hand, resulted in a discretely lower expression of the mRNA of the *fmo3* gene as well as lower levels of the FMO3 enzyme and TMAO. Hence, both androgens/dihydrotestosterone and estrogens seem to affect the formation of FMO3 and TMAO [43].

## 4. Molecular TMAO Mechanisms in Host Cells

TMAO is known to participate in multiple biological functions. For example, it acts as a protein stabilizer, preserving its structure and enzymatic activity as well as counteracting the effects of pH, urea, and increased pressure [6]. 

### 4.1. Cardiovascular and Endothelial Cells

TMAO alters several mechanisms and/or pathways related to different human diseases. The signaling pathways of the immune system play a key role in such mechanisms. Smad3 is a signaling transducer involved in the cascade induced by transforming growth factor beta (TGF-β). The TMAO-induced stimulation of the Smad3 signaling pathway antagonizes the inhibitory effect of this metabolite on cell growth and elicits cardiac hypertrophy and fibrosis. These effects have been found through in vitro studies with cardiomyocytes and in a rat model [44]. Another immunity-related mechanism is the TMAO-mediated inflammation triggered by damage to the endothelial cells. This damage leads to the TMAO-dependent activation of the NLRP3 inflammasome and the inflammatory cytokines interleukin (IL)-1β and IL-18 together with the inhibition of nitric oxide synthase [45].

In murine models, a diet that is rich in the TMAO precursors (e.g., choline) was reported to increase the expression of MCP1 (monocyte chemotaxis protein 1), MIP-2 (macrophage inflammatory protein 2), TNF (tumor necrosis factor), ICAM1 (intercellular adhesion molecule 1), KC (keratinocyte-derived chemokine), Cox-2 (cyclooxygenase 2), E-selectin, VCAM1 (vascular cell adhesion protein), and the macrophage marker CD68 in the aortic tissue. In one study, an injection of TMAO into endothelial cells induced the generation of p38 mitogen-activated protein kinase, extracellular signal-related kinase 1/2, and p65 NF-kB, which are all important signaling molecules for promoting cellular inflammation. The NF-kB activation is needed to trigger an exaggerated inflammation response in the endothelial cells, which would favor leukocyte adhesion [46]. Furthermore, an increased serum TMAO concentration was associated with a greater expression of CD36 (a renowned scavenger receptor) in the macrophages, which led to the internalization of lipids, the formation of foam cells, and the development of atherosclerosis in ApoE −/− mice [47].

Additionally, in ApoE −/− knockout mice receiving a diet rich in TMAO precursors, there was an upregulation of the B subunit of the succinate dehydrogenase complex. Pyroptosis was found in vascular endothelial cells and in cultured human umbilical vein endothelial cells treated with TMAO [48].

TMAO induced the senescence of the human umbilical vein endothelial cells (HUVEC cells) in vitro, evidenced by an increased presence of senescence markers, reduced cell proliferation, G0/G1 stalling, and impaired cell migration. In addition, TMAO repressed the production of sirtuin-1 (SIRT1), and the resulting greater oxidative stress affected the p53 pathway [49]. Hence, TMAO contributes significantly to the senescence of endothelial cells through oxidative stress.

In addition, TMAO is capable of triggering oxidative stress. In aged rats, it caused an increased production of proinflammatory cytokines and superoxide as well as a decrease in the endothelial nitric oxide synthase molecules in the aorta. The level of these molecules can be restored through treatment with 3,3-dimethyl-1-butanol, an inhibitor of TMA synthesis [30].

### 4.2. Immune Cells

The exposure of J774A macrophages to TMAO leads to the overexpression of TLR4 receptors, which promotes the production of proinflammatory cytokines [50]. TMAO also gives rise to an elevated level of two endoplasmic reticulum stress markers in J774A macrophages, HSP60 and GRP78. Hence, TMAO could potently cause endoplasmic reticulum stress and could activate the unfolded protein response (UPR) pathway [51].

### 4.3. Hepatocytes

In the hepatocytes, TMAO stimulates the production of exosomes, thus altering gene expression in the aortic endothelial cells. Considering the resulting differential expression of the genes, the enriched functions are associated with cytokine-receptor-linked signaling in the immune system, extracellular-matrix-linked proteins, and interferon signaling [52]. Among these genes, the CXCR4 gene has been identified as a key gene since its negative regulation, induced by TMAO exosomes, significantly diminishes cell migration and angiogenesis. Such effects can be reversed through the overexpression of CXCR4 [52]. 

### 4.4. Kidney Cells

TMAO also induces endoplasmic reticulum (ER) stress in the cell line of HEK293 cells through the activation of PERK and FoxO1, leading to potential insulin resistance and, thus, increasing the risk of developing hyperglycemia, metabolic dysfunction, and type 2 diabetes in patients with higher levels of TMAO [53]. The insulin receptor INSR is one of the downstream target genes of FoxO1 and is upregulated through PERK activation, inducing cellular insulin resistance [54].

### 4.5. Cancer Cells

TMAO was also reported to affect protein folding [55], and it was more recently reported to alter the cis–trans isomerization of proline and to prevent the folding of an enzyme called carbonic anhydrase [56]. In detail, TMAO induces misfolding/unfolding at the cellular level in HeLa cells, thus disrupting the cell cycle, restricting cells to the S phase, and impairing cell division. The accumulation of misfolded proteins in the endoplasmic reticulum can promote a stress reaction through the UPR, which, together with the TMAO-induced upregulation of inflammatory markers, may favor a proinflammatory response [56]. 

### 4.6. Neurons

Another type of TMAO-induced change has been detected in the neurons. Alterations in the synapses and in neuronal plasticity detected in ex vivo models generate endoplasmic reticulum stress in the hippocampal neurons through the activation of the PERK pathway. A modification of presynaptic receptors and a decreased expression of postsynaptic receptors were also observed [57].

### 4.7. Platelets

The TMAO role as a platelet activator was demonstrated through the amelioration of the effects of clopidogrel on platelet aggregation in a murine model [58]. The mechanism of TMAO-induced platelet hyperreactivity is mediated through platelet activation, which is dependent on multiple agonist stimuli via calcium ions [59].

Notably, almost all the proteins linked to the inflammatory response mentioned in this section are thought to participate in the pathogenesis of several diseases (Table 3). Thus, as discussed in detail in next section, TMAO could play an important role in diseases such as diabetes, chronic kidney disease, obesity, cardiovascular diseases, cancer, and neurological diseases.

## 5. Human TMAO-Associated Diseases

**Figure 2:**
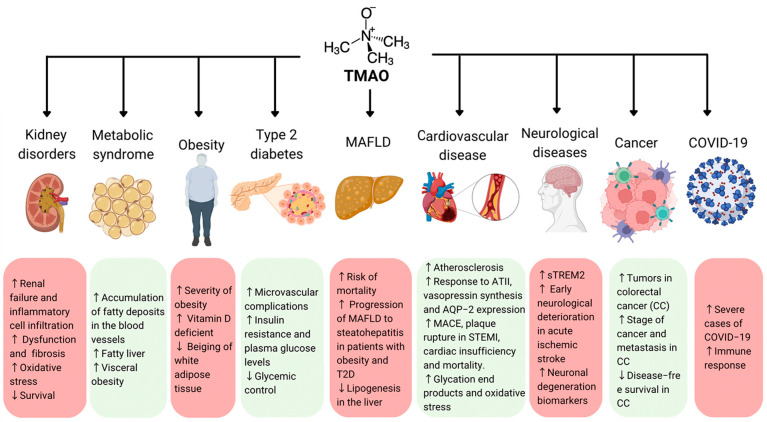
The role of TMAO in human health. TMAO has been associated with an enhanced immune response, which is involved in the pathogenesis of several diseases, such as cardiovascular and neurological diseases. An elevated level of TMAO has been linked to kidney disorders; metabolic syndrome; obesity; type 2 diabetes; MAFLD; cardiovascular diseases; neurological diseases; cancer; and, recently, severe cases of COVID-19. Arrows indicates increased (upper arrow) or decreased processes (lower arrow) (Created with canva.com and BioRender.com).

As previously reported, TMAO is a biomolecule capable of providing relevant information on the metabolic and immunological state of the human body. Having been linked to the pathogenesis and progression of several diseases (Figure 2), it could be a potential biomarker for diagnosis, prognosis, and therapeutic intervention.

### 5.1. Kidney Disorders

Although 95% of TMAO is normally eliminated by the kidneys, this filtering function is affected by chronic kidney disease. Interestingly, TMAO was found to be a marker of survival in patients with chronic kidney disease in a prospective study using the glomerular filtration rate, C-reactive protein, and cystatin C as its evaluation parameters [60].

TMAO’s role in chronic kidney disease has also been examined in animal models. For instance, a decrease in the plasma concentration of TMAO attenuated the progression of chronic kidney disease [32]. In addition, TMAO has been reported to accelerate the development of diabetic kidney disease, exacerbating renal dysfunction and fibrosis by activating the NLRP3 inflammasome and by promoting the release of IL-1β and IL-18 [61]. Similarly, renal failure and inflammatory cell infiltration were exacerbated by TMAO treatment in CDK rats, leading the authors to conclude that this microbial metabolite made inflammation more robust. The cytokines MCP-1, TNF, IL-6, IL-1β, and IL-18 were activated through the p38 pathway. Oxidative stress was increased through the upregulation of NOX-4, the downregulation of SOX, and the activation of the NLRP3 inflammasome by caspase-1 and IL-1β [62]. 

In addition, research has been performed on patients with chronic kidney disease. The TMAO level was higher in patients with chronic kidney disease than in the control group, and it was highest in patients with declining renal function. In detail, the concentration of TMAO correlated negatively with the glomerular filtration rate and positively with the levels of IL-6 and fibrinogen. Moreover, an elevated TMAO level was associated with a drop in the 5-year survival and with a 6.3-fold increase in the risk of mortality [63].

The level of TMAO being inversely correlated with the glomerular filtration rate explained the higher level. Moreover, the FMO3-induced production of TMAO may be another factor contributing to the increased TMAO level. The renal elimination of TMAO was only slightly affected by tubular secretion and reabsorption, suggesting that the goal of reducing the TMAO level must be achieved by targeting its production [64].

Overall, the aforementioned reports provide the basis for proposing TMAO as a therapeutic target. However, more research is needed on whether a decreased plasma concentration of TMAO influences the glomerular filtration rate. In a double-blind randomized study, an evaluation was performed on the use of probiotics for lowering the concentration of TMAO and for improving kidney function, finding no significant difference with the placebo group [65]. Therefore, it is necessary to investigate the long-term effect of probiotics together with dietary intervention as a strategy for diminishing the amount of TMAO and for preserving renal function.

### 5.2. Metabolic Syndrome

Metabolic syndrome (MS) involves at least three of the following five conditions: abdominal obesity, systemic arterial hypertension, insulin resistance, a high serum concentration of triglycerides, and a low serum concentration of HDLs (high-density lipoproteins). MS is a risk factor for the development of type 2 diabetes and cerebrovascular and cardiovascular diseases [66]. The presence of a high serum concentration of TMAO has been associated with metabolic syndrome, probably due to the multiple pathways of inflammation in which this metabolite participates, including the accumulation of fatty deposits in the blood vessels and tissues, which generates a fatty liver, visceral obesity, and atherosclerosis [67].

According to the results of a transversal study, a high serum concentration of TMAO (≥8.74 µM) was proposed as a biomarker for MS, as it implies a high risk of developing this disorder [68]. Additionally, more details about the diseases related to metabolic syndrome are mentioned in the following sections. 

### 5.3. Obesity

Obesity is a worldwide public health problem and is defined as excessive fat accumulation in the body. There has been a 5-fold increase in the number of obesity cases since 1997 [69]. Obesity is a risk factor for several chronic noncommunicable diseases, including cardiovascular diseases and diabetes [70]. 

An elevated TMAO level is linked to obesity and a vitamin D deficiency, although the causality has not been established [71]. Surely the diet is an important factor in the connection between TMAO and obesity. In a random clinical trial of 62 participants with overweight and obesity factors, 3 groups were formed and observed over 8 weeks: a control group, a cod diet group, and a salmon diet group. The level of TMAO was higher in the cod diet group than the salmon diet or control groups [72]. 

TMAO accumulation has been proven to be unaffected in the short run by other foods, such as eggs (in postmenopausal women) or those containing flavonoids (in obese patients) [73,74]. In a study on obese adults, the level of TMAO was lower in participants assigned exercise in combination with a hypocaloric diet (<500 kcal) compared to those with an equicaloric diet. When developing interventions that seek to decrease the level of TMAO in overweight or obese patients, it is needed to consider the bone mineral loss of the spine, which is reportedly independent of changes in body weight [75]. A systematic review found a nonlinear relationship between the TMAO level and the body mass index (BMI) among 56,556 apparently healthy patients [76].

Finally, the mechanisms of TMAO in obesity progression have been examined in animal models. The deletion of FMO3, the enzyme responsible for the conversion of TMA into TMAO, conferred protection against obesity in mice by allowing for the begging of white adipose tissue [77].

### 5.4. Diabetes

Diabetes is a chronic metabolic disease characterized by an elevated blood glucose level that is capable, over time, of leading to damage to the heart, blood vessels, eyes, and kidneys. The most common form of this disease in adults is type 2 diabetes, which begins with insulin resistance and may progress to a low insulin level. In the last 30 years, the incidence of diabetes has been growing rapidly around the world [78].

As was mentioned before, TMAO can induce ER stress through PERK and FoxO1 activation, leading to the expression of INSR and insulin cellular resistance and elevating the risk of developing type 2 diabetes [53]. There are several clinic studies that confirm the association between TMAO and type 2 diabetes. In one study, the serum concentration of TMAO was higher in T2D patients compared to those with prediabetes or without diabetes [79].

In the POUND LOST trial, a minor reduction in the TMAO level was linked to small improvements in the blood concentration of glucose and insulin as well as in the degree of insulin resistance (the latter was evaluated with the homeostatic model assessment, HOMA) in participants who consumed a high-fat hypocaloric diet [80]. There was a relationship between TMAO, T2D, and its complications. It has been hypothesized that microvascular endothelial damage is produced by both the disease and by TMAO [81,82].

In one study, 4442 participants from a cohort of older US adults from the Cardiovascular Health Study (CHS) were observed for 7 years, finding no association between TMAO and the incidence of T2D. Nevertheless, the concentration of TMAO showed a positive association with the fasting insulin concentration, a marker of insulin resistance [83].

Experimental work using animal models demonstrated the effects of TMAO on the glucose metabolism. Glucose tolerance was affected in the insulin signaling pathway of the liver, increasing the synthesis of proinflammatory mediators in the adipose tissue [84]. Moreover, a decrease in the TMAO level achieved through the elimination of FMO3 led to a lower plasma concentration of glucose and insulin. Contrarily, a high amount of FMO3 increased insulin resistance and the plasma concentration of glucose [85].

### 5.5. Metabolic-Dysfunction-Associated Fatty Liver Disease

Metabolic-dysfunction-associated fatty liver disease (MAFLD), previously denominated nonalcoholic fatty liver disease, is an inflammatory condition that is closely linked to the level of TMAO as well as to MS, T2D, and obesity. There is a close relationship between adipose tissue dysfunction (characterized by increased cytokine/chemokine production as well as an influx of CD4^+^ macrophages, CD8^+^ T cells, dendritic cells, and natural killer cells) and insulin resistance in the liver, muscle, and adipose tissues [86]. MAFLD should be promptly identified because it usually progresses to more serious forms, including steatohepatitis, steatofibrosis, cirrhosis, and cancer [87]. It is also associated with the development of liver cancer as well as esophageal, gastric, uterine, colon, breast, and other cancers [88]. Indeed, MAFLD is linked to a greater risk of mortality from any cause [89].

The relationship between TMAO and the progression from MAFLD to steatohepatitis has mainly been described in obese patients with T2D [90]. This relationship owes itself to the capacity of TMAO to promote de novo lipogenesis in the liver, which increases the generation of bile acid and inhibits farnesoid X receptor activation [91]. Hence, TMAO has been proposed as a possible therapeutic target for MAFLD [92].

### 5.6. Systemic Arterial Hypertension

Systemic arterial hypertension (SAH) is highly prevalent in adults and represents the main risk factor for cardiovascular and cerebrovascular diseases. In the respective patients, an elevated serum TMAO concentration is associated with an atherosclerotic effect and an overabundance of proinflammatory cytokines [93]. In animal models, a high level of TMAO prolonged the hypertensive response to angiotensin II, which increases vasoconstriction and the concentration of the intracellular ionic calcium in the arterioles [94]. It also caused a greater synthesis of vasopressin, expression of acuaporin-2, and osmotic pressure [95].

In mice, the TMAO concentration was associated with age and was positively correlated with a higher systolic blood pressure (SBP) and arterial stiffness independently of other cardiovascular risk factors. An elevated TMAO level is associated with more abundant amount of advanced glycation end products in the aorta, and in ex vivo experiments, it boosted the SBP and exacerbated arterial stiffness via the glycation end products and oxidative stress [96]. In another animal model, the treatment of obstructive-sleep-apnea-induced hypertension in rats with the probiotic *Lactobacillus rhamnosus* GG strain provided a lower level of TMAO and CD4^+^ T cell induced-type I inflammation, leading to a reduction in hypertension [97].

### 5.7. Vascular Diseases

**Figure 3:**
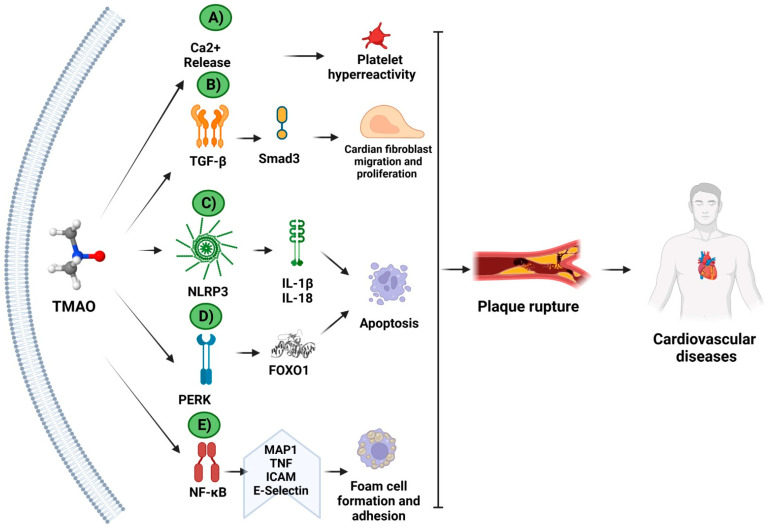
Molecular mechanisms of TMAO in cardiovascular diseases. The mechanism of TMAO in cardiovascular diseases has been studied in animal and/or cellular models. TMAO increases the (**A**) Ca^2+^ release and platelet hyperactivity; (**B**) TFG-β activation and Smad3 signaling pathway; (**C**) NLRP3 inflammasome activation, inducing apoptosis; (**D**) PERK-unfolding protein response (UPR) activation and endoplasmic reticulum stress; and (**E**) NF-κB translocation and foam cell formation and adhesion. Together, these mechanisms contribute to plaque ruptures, and, therefore, to heart disease. (Created with BioRender.com).

The effects of an elevated TMAO level have most commonly been analyzed in relation to cardiovascular and cerebrovascular diseases. TMAO has been evaluated as a biomarker of major adverse cardiovascular events (MACEs), cardiac insufficiency, and mortality by vascular disease [98,99,100]. The mechanism of TMAO in cardiovascular diseases is shown in Figure 3.

In an observational study, an association became apparent between a two-fold increase in TMAO and a higher risk of mortality, sudden cardiac death, one’s first cardiovascular event, and death in a Caucasian population [101]. Several other research efforts have proposed TMAO as a biomarker for cardiovascular diseases. The monitoring of patients in Japan demonstrated a positive correlation between the level of TMAO and the number of infarcted coronary arteries in patients who underwent a cardiovascular surgery [102]. According to an investigation in China, a high TMAO concentration is linked to plaque ruptures in hospitalized patients with ST-segment elevation myocardial infarction (STEMI) [103]. In Thai patients with subclinical myocardial damage, there was a positive correlation between a greater level of TMAO and high-sensitivity cardiac troponin [104]. In a study carried out in the USA (Cleveland, Ohio), TMAO proved to be a good biomarker for the prediction of MACEs, even in patients with a negative troponin. Hence, the use of TMAO as a potential biomarker in cardiovascular diseases goes beyond its mere use in the usual tests for the diagnosis of acute coronary syndrome [105].

Other reports have shown a positive linear correlation between a high serum TMAO concentration and the risk of cerebrovascular diseases [106] as well as with its recurrence within one year [107]. One article described a higher risk of one’s first cerebrovascular accident and the worsening of neurological deficits in patients with cerebrovascular diseases who had an elevated amount of TMAO [108]. Compared to patients with atrial fibrillation and ischemic stroke, the level of TMAO was lower in patients with atrial fibrillation alone [109]. According to all these results, TMAO may serve as a biomarker of risk for patients with acute coronary syndrome and other cardiovascular diseases. This indicator would be independent of the traditional factors for cardiovascular diseases, such as hypertension, dyslipidemia, and smoking. 

Medical interventions have been utilized to evaluate a potential effect on the TMAO levels of patients. For instance, 100 mL of Sanhuang Xiexin (a traditional Chinese formula) was administered 2 times per day for 1 week to 121 patients with an acute ischemic cerebrovascular disease under standard treatment. This prospective observational study found that the level of TMAO was lower in 61 of the patients in the treated group than in those with no administration. In addition, the risk of ischemic events was also lower in the Sanhuang Xiexin group versus the control group during the third and sixth months [110]. 

TMAO activity in the development of cardiovascular diseases has been assessed in vitro. The exposure of the cardiac mitochondria of rats to TMAO impaired β-oxidation and decreased the pyruvate metabolism. Thus, an elevated TMAO level could be considered to be a risk factor for cardiovascular events due to the disturbance of the energy metabolism in the cardiac tissue [111].

Probiotics were consumed by male patients with stable coronary artery disease for six weeks, finding improved endothelial function and reduced inflammation, but the TMAO concentration remained the same [112]. In another attempt to diminish the level of TMAO, an autologous fecal transplant was performed on patients with metabolic syndrome using tissues from a vegan donor. This intervention resulted in changes in the composition of the gut microbiota but not in the level of any measured parameter related to vascular inflammation, including TMAO [113].

Finally the resveratrol is a natural polyphenol contained in grapes, berries, and other foods, and it is used for the treatment of several metabolic diseases, such as atherosclerosis [114]. Its consumption reportedly modulates the composition of the gut microbiota, increasing the *Bacteroidetes*–*Firmicutes* ratio and the growth of *Bacteroides*, *Lactobacillus*, and *Bifidobacterium*. When ApoE −/− mice (a murine model of atherosclerosis) were treated with choline and then resveratrol, TMAO synthesis was blocked. Since FMO3 production was upregulated, the effect of reducing the TMAO level did not occur in the liver due to FMO3 but rather due to the increase in the *Bacteroides*–*Firmicutes* ratio and the growth of *Lactobacillus* and *Bifidobacterium*, which was evidenced by a lower bacterial content in the ileum and the repression of the farnesoid X receptor. Thus, resveratrol attenuated TMAO-induced atherosclerosis by decreasing the TMAO levels and by modulating the gut microbiota composition [115].

Further research is needed on probiotics and other treatments aimed at diminishing the TMAO concentration and at lowering the risk factors associated with cardiovascular diseases.

### 5.8. Neurological Diseases

**Figure 4:**
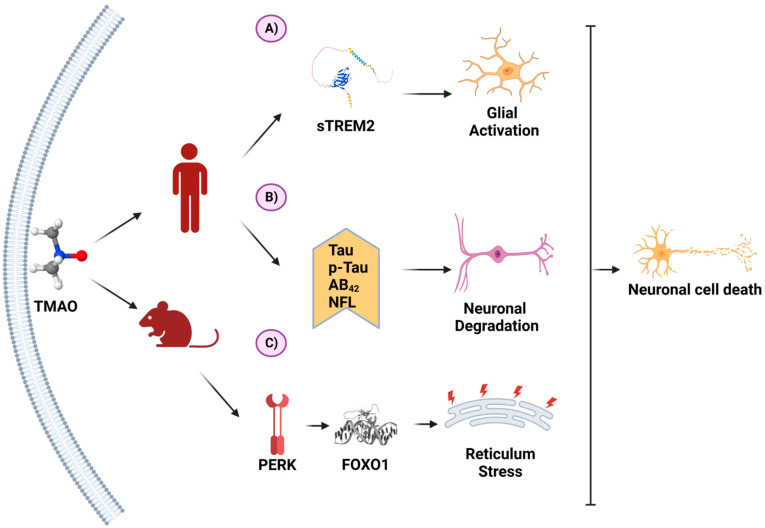
TMAO and its molecular mechanisms in neurons. (**A**) In a clinical study on patients with Alzheimer’s disease (AD), TMAO was positively associated with sTREM2, a biomarker for glial activation. (**B**) In another clinical study on AD, TMAO was associated with higher levels of AD biomarkers. (**C**) In an animal model of AD, TMAO induced the unfolding protein response through PERK activation, inducing endoplasmic reticulum stress. These processes favor neuron degeneration and death. (Created with BioRender.com).

Recently, TMAO has been reported to exhibit neuroinflammatory activity. The proposed mechanism is shown in Figure 4.

A relationship was detected between the intake of red meat or cheese and the level of carnitine in human cerebrospinal fluid samples. Moreover, there was a positive correlation between TMAO and sTREM2, a glial activation marker [116].

In patients with acute ischemic stroke, high TMAO levels were linked to early neurological deterioration. The probable explanation is that several inflammatory markers, such as C-reactive protein and IL-6, are associated with the progression of neurological deficits. Since TMAO is able to trigger an exacerbated inflammatory response, it can likely cause the worsening of neurological deterioration in such patients [117].

The use of ApoE −/− mice to study the connection between TMAO and atherosclerosis was mentioned in the previous section on vascular diseases. ApoE is expressed by several cellular types but is mainly found in some immune cells of the central nervous system: hepatocytes, astrocytes, and microglia. In these cells, it plays an important role in the metabolism of plasma lipoproteins and in the growth and maturation of the neurons. 

The *apoE* gene has been identified as a genetic risk factor for atherosclerosis, cardiovascular diseases, and Alzheimer’s disease (AD). The ε4 allele of the *apoE* gene increases the risk of Alzheimer’s disease, while the ε2 allele provides protection against it [118]. The carriers of the ε4 allele also have a higher risk of hypercholesterolemia and atherosclerosis because this allele leads to a greater plasma concentration of LDLs [119]. In addition, the ApoE protein interacts with the beta-amyloid peptide. The latter, one of the main elements in AD pathogenesis, is responsible for neural death and brain degeneration.

A critical factor of AD progression is an exaggerated inflammatory response (as is the case with atherosclerosis as well), and TMAO promotes inflammation and oxidative stress. Hence, an elevated level of this microbial metabolite could help to trigger the exacerbated inflammatory response that accompanies Alzheimer’s disease, an idea reinforced by the altered gut microbiota found in AD patients. Such an alteration is mainly manifested as a decreased abundance of *Firmicutes* and *Bifidobacterium* and an increased abundance of *Bacteroidetes*, which are genera linked to TMA production. The proportion of these genera in the microbiota composition is also modified in patients with cardiovascular diseases [120].

Given that inflammation plays an important role in Alzheimer’s disease, it is not surprising that several immune molecules have been linked to its pathogenesis, including TLR4, TGF-β, CXCR4, MCP-1, MIP2, Cox-2, NLRP3, PERK, and FoxO1. As previously stated, in vitro studies have shown that the aforementioned proteins are activated or induced by TMAO.

There are some reports on the level of TMAO in AD patients. In detail, a transversal study documented a higher level of TMAO in the cerebrospinal fluid of AD patients with mild cognitive deterioration than in that of those with no cognitive impairment. When comparing patients with severe dementia to those with a mild cognitive deterioration, higher TMAO amounts were exhibited by the former. Furthermore, TMAO was positively correlated with Alzheimer’s disease and with the biomarkers of neuronal degeneration (p-tau, p-tau/AB_42_, total tau, and neurofilament light chain protein) [121]. 

In animal models, an elevated level of TMAO has been positively correlated with more pronounced AD symptoms, such as long-term potentiation, increased neuronal loss, and altered spatial learning and memory [122]. Thus, AD patients might experience relief of their symptoms by means of interventions capable of modifying the concentration of TMAO in circulation, which could be accomplished in part by reducing the elements of the gut microbiota responsible for activating the TMAO pathway. The corresponding treatments would be expected to modulate the inflammatory response and to attenuate the neuronal deterioration of patients suffering from Alzheimer’s disease.

### 5.9. Cancer

In recent years, TMAO has been analyzed as a risk factor for cancer, especially colorectal cancer. Several studies have demonstrated that an altered microbiota composition is associated with colorectal cancer (CRC), which involves a greater abundance of the species of the genus *Fusobacterium* [123]. The degree of TMAO production depends on the composition of the gut microbiota.

The first report of a TMAO association with colorectal cancer was based on a genome-wide systemic analysis conducted in 2015, which established the genetic relationship between TMAO and several diseases, including cardiovascular diseases, metabolic syndrome, and some cancer types such as colorectal cancer [124].

Subsequently, research was carried out on patients to explore the possible link between TMAO and colorectal cancer. The first example was the Women’s Health Initiative Observational Study, finding a positive correlation between TMAO and female CRC patients. In the same study, the patients with higher TMAO levels showed a 1.9-fold greater risk of proximal tumors, a 2.3-fold greater risk of rectal tumors, and a 1.8-fold greater risk of local tumors. These risks were associated with a low level of vitamin B12 [125].

In another study performed on male patients in Finland, the alpha tocopherol and beta carotene cohorts showed no association between colorectal cancer and TMAO. Nevertheless, choline (one of the precursors of the TMAO biosynthesis pathway) was associated with colorectal cancer [126].

In China, TMAO concentrations were evaluated for 108 patients (68 men and 40 women), finding a significantly higher level in CRC patients than in the healthy controls. Regarding an elevated level of TMAO, there was a positive correlation with a more advanced CRC stage and with the presence of metastases as well as a negative correlation with disease-free survival. Hence, the results pointed to TMAO as a plausible prognostic marker for colorectal cancer [127].

By utilizing bioinformatics, TMAO has been linked to the development of other cancers, such as ovarian, breast, and gastric cancers. However, such TMAO activity has not been fully explored [124]. Besides colorectal cancer, only prostate cancer has been directly linked to TMAO [128].

Although the mechanisms linking TMAO to different cancers are not completely understood, they likely involve the proinflammatory potential of this microbial metabolite. As explained herein, TMAO is able to trigger an inflammatory response through several pathways, including the production of inflammatory markers regulated by NF-kB, Smad3, NLRP3, and the unfolded protein response in J774A macrophages [44,45,46,51].

It has also been proposed that TMAO increases the risk of cancer through oxidative stress, which damages DNA when antioxidant defenses are deficient. In addition, higher levels of reactive oxygen species are found in cancer versus healthy cells, thus contributing to the sustenance of the malignant cell phenotype [129]. Therefore, the TMAO ability to induce oxidative stress may favor the growth and proliferation of cancer cells, leading to cancer development or the worsening of its clinical symptoms.

Another risk factor where TMAO is involved is the induction of insulin resistance. Obesity, insulin resistance, and type 2 diabetes are risk factors of developing cancers such as endometrial, pancreatic, liver, and colorectal cancers [130]. They can be explained by changes in the expression of insulin receptors [131], and, as is mentioned in a previous section, these receptors can be induced by TMAO through PERK/FoxO1 activation. IRS-1 and IRS2 receptors are critical for colorectal cancer prognoses, where both receptors are associated with metastases [132,133].

### 5.10. COVID-19

Coronavirus disease (COVID-19) is an infectious disease caused by the SARS-CoV-2 virus. It first appeared in China in 2019 and then spread rapidly around the world, creating a pandemic with millions of cases of infection and death. Although most people infected with the virus only experience mild respiratory symptoms and recover without medical intervention, the symptoms can be life-threatening and can require advanced medical care [134]. 

The risk factors for severe COVID-19 are underlying medical conditions such as cardiovascular diseases, diabetes, chronic respiratory diseases, and cancer. SARS-CoV-2 activates a stress pathway in the host immune system, promoting an aberrant response in the adaptive and innate immune systems and probably leading to pneumonia and death [135]. As aforementioned, TMAO has an impactful role in the pathogenesis of cardiovascular diseases and diabetes, which are known risk factors for severe COVID-19. Moreover, like COVID-19, TMAO is able to modulate the immune response. Hence, it has been suggested that TMAO contributes to the COVID-19 pathogenesis.

Evidence exists of an interaction between SARS-CoV-2 and the NLRP3 inflammasome which occurs to increase IL-1 and IL-18 production through NF-ҡB activation [136]. Some studies on SARS-CoV-2-infected patients pointed to a relationship between TMAO and the development of severe cases of COVID-19. For example, an alteration in the gut microbiota was reported in COVID-19 patients (compared to healthy subjects). Whereas the relative abundance of *Coprobacillus, Clostridium ramosum,* and *Clostridium hathewayi* was correlated with the COVID severity, *Faecalibacterium prausnitzii* correlated negatively with the same factor [137]. Both the *Coprobacillus* and *Clostridium* species contain the *cutC* gene, while the *Clostridium* species also bares the *grdH* gene according to the UNIPROT database (www.uniprot.org). Thus, it is highly probably that both *Coprobacillus* and *Clostridium* contribute to TMA production.

When TMAO and its precursors were measured in patients with COVID-19, a low level of betaine was associated with a poor outcome. Hence, this metabolite was proposed as a biomarker of the risk factors for COVID-19. However, no significant difference was detected between the TMAO levels of COVID-19 patients versus those of the healthy controls [138]. The study served as a proof of concept, meaning that more research is required to clarify the connection between TMAO and COVID-19. 

## 6. Conclusions

There is robust evidence of the association between high levels of TMAO and multiple diseases, including cardiovascular and kidney diseases, cancer, and Alzheimer’s disease. In the pathogenesis of all these diseases, the inflammatory response is a key mechanism. Given that TMAO is able to regulate the inflammatory response and that previous reports documented the participation of the intestinal microbiota in the gut–brain axis, TMAO may be implicated in immunity modulation in these diseases. It is necessary to gain greater insights into the pathogenic TMAO mechanisms and the most effective treatment approaches to prevent potential complications.

Since multiple factors influence the level of TMAO, integral interventions involving diet and probiotics may improve the quality of life of subjects at a high risk for developing chronic inflammatory and degenerative diseases. Specifically, advances in genetic engineering should be instrumental in the design of probiotics that are capable of decreasing the production and availability of the TMAO precursors. One of the recurrent themes in the relationship between TMAO and various diseases is the change in microbiota composition and function. Consequently, it is crucial to propose intervention protocols requiring microbiota shaping. Finally, TMAO could become a potential biomarker for the diagnosis of high-impact diseases in the general population.

## Figures and Tables

**Table 1:** Bacterial genes related to TMAO metabolism.

Gene	Protein Name	Function
TMA-producing genes		
*cutC*	Choline lyase	Cleaves the C-N bond in choline, producing TMA
*cutD*	Choline trimethylamine-lyase activating enzyme	Activates *cutC*
*cntA*	Carnitine monooxygenase	Converts carnitine to TMA
*cntB*	Carnitine monooxygenase reductase	Converts carnitine to TMA
*yeaW*	Carnitine monooxygenase	Converts carnitine to TMA
*yeaX*	Carnitine monooxygenase reductase	Converts carnitine to TMA
*grdH*	Betaine reductase	Converts betaine to TMA
TMAO respiration		
*torC*	C-type cytochrome	Final electron acceptor in anaerobic respiration
*torA*	TMAO-reductase	Reduces TMAO to TMA
*torD*	torA chaperone	torA chaperone
Methane production from TMA		
*MttB*	TMA methyl transferase	Transfers the methyl group in TMA

**Table 2:** Drugs/compounds, and their origins and mechanisms, that reduce TMAO level.

Drug/Compound	Origin	Mechanism	Reference
Statins	Drugs used to treat atherosclerosis	Gut microbiota alterations	[18,19]
Metformin	Drug used to treat type 2 diabetes	*Cut* gene cluster proteins	[22]
Aspirin	NSAID	Gut microbiota alterations, inhibition of *cutC* TMA-lyase	[26,27]
Vitamin D	Fat-soluble vitamin	Gut microbiota alterations	[35]
Berberine	*Coptidis Rhizoma*	Inhibition of *cutC* TMA-lyase	[28]
3,3-Dimethyl-1-butanol (DMB)	Balsamic vinegar, red wine, olive oil, and grape seed oil	Inhibition of *cutC* TMA-lyase	[29]
Meldonium	Anti-ischemic drug	Inhibition of *cntA* monooxygenase	[33]
Iodomethylcholine (IMC)	Synthetic halomethylcholine analogue	Inhibition of *cutC* TMA-lyase	[34]
Fluoromethylcholine (FMC)	Synthetic halomethylcholine analogue	Inhibition of *cutC* TMA-lyase	[34]
Benzoxazole ligand (BO-I)	Benzoxazole derivative	Inhibition of *cutC* TMA-lyase	[36]
Betaine aldehyde	*E. coli*, plant, *Homo sapiens*, and *Aspergillus fumigatus* metabolite	Inhibition of *cutC* TMA-lyase	[37]

**Table 3:** The effect of TMAO on disease-related signaling pathways.

Mechanism	Molecules and Pathways		Associated Diseases							
			CKD	MS	Obesity *	T2D *	MAFLD *	CAD	NDs *	Other
Proliferation, migration, and collagen secretion	TLR4	CXCR4		Yes	Yes	Yes	Yes		Yes	Cardiac Fibrosis
	TGF-β/Smad3									
Endothelial dysfunction	MCP-1 ^c^ MIP-2 ^b,c^ TNF-α ICAM1 ^a^ VCAM1 ^a^	CXCL1 ^a^ Cox-2 E-selectin ^a^ NF-kB NLRP3	Yes	Yes	Yes	Yes	Yes	Yes	Yes	
	CD36 and CD68 receptors									
Platelet aggregation	Ca2+ release		Yes	Yes		Yes		Yes	Yes	CRC
RE stress	PERK FOX01	HSP60 GRP78		Yes	Yes	Yes	Yes		Yes	
Apoptosis	NLRP3	TGF-β			Yes	Yes	Yes	Yes	Yes	Cancer
	Sirtuin 1									

CXCR4 represents C-X-C motif chemokine receptor 4, MCP1 represents monocyte chemoattractant protein-1, MIP2 represents macrophage inflammatory protein-2, TLR4 represents Toll-like receptor 4, TGF-β represents transforming growth factor beta, Smad3 represents mothers against decapentaplegic homolog 3, TNF-α represents tumor necrosis factor-alpha, ICAM-1 represents intercellular adhesion molecule 1, KC represents keratinocyte-derived chemokine, VCAM 1 represents vascular cell adhesion protein, CXCL1 represents C-X-C motif chemokine ligand 1, PERK represents protein kinase RNA-like endoplasmic reticulum kinase, FoxO1 represents forkhead box protein O1, NLRP3 represents NLR family pyrin domain containing 3. a = not described in chronic kidney disease, b = not described in cancer, c = not described in metabolic-dysfunction-associated fatty liver disease. * = disease associated with a high level of TMAO in which the mechanism has been described without verifying that TMAO affects the corresponding molecules. Chronic kidney disease (CKD), metabolic syndrome (MS), type 2 diabetes (T2D), metabolic-dysfunction-associated fatty liver disease (MAFLD), coronary arterial disease (CAD), neurological diseases (NDs), and colorectal cancer (CRC).

## Data Availability

Not applicable.
